# Supplementary material for: Insights from Syzygium aromaticum Essential Oil: Encapsulation, Characterization, and Antioxidant Activity
Source: Pharmaceuticals (Basel). 2024 May 8;17(5):599. doi: 10.3390/ph17050599 (PMC11124181; doi:10.3390/ph17050599)
Supplement: Supplementary file 1 [file pharmaceuticals-17-00599-s001.zip › pharmaceuticals-2987808-supplementary.pdf]

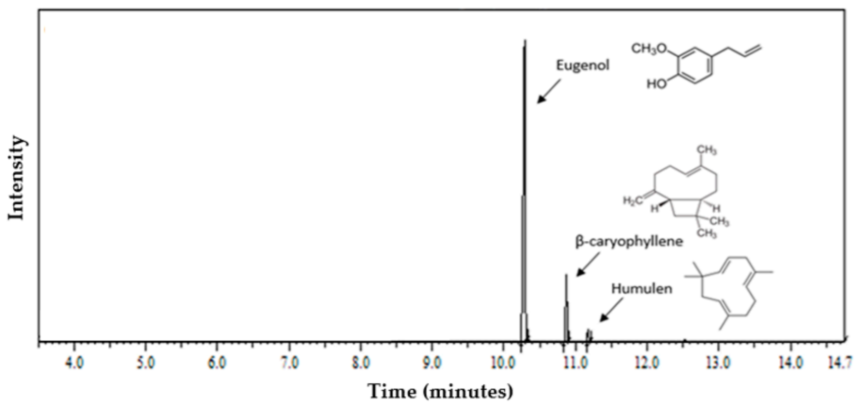

Figure S1. Chromatographic profile of clove essential oil obtained by GC/MS.

Table S1: Main thermogravimetric and calorimetric data of the samples.

Samples		Thermogravimetry				Scanning Calorimetry		
		TR (°C)	Midpoint (°C)[1]	Weight loss (%)		TR (°C)	PM (°C)[1]	ΔH (J/g)[2]
**CEO**	I	161.3 – 201.1	179.2	99.0	I	25.0 – 44.9	26.9	-38.3
					II	164.1 – 180.0	176.9	-85.2
					III	261.3 – 281.1	272.9	-54.5
					IV	397.8 – 427.6	365.9	+188.3
**LF1**	I	63.0 – 120.9	86.8	68.4	I	71.7 – 95.2	71.6	+2.8
	II	207.0 – 240.9	218.8	12.1	II	98.4 – 115.9	103.8	-9.9
	III	299.7 – 336.5	314.4	9.2	III	193.3 – 215.9	258.2	-1.7
					IV	386.6 – 420.2	319.5	-35.6
**LF2**	I	63.2 – 106.4	83.1	45.1	I	122.6 – 134.3	123.2	-224.4
	II	211.4 – 239.7	224.0	6.6	II	297.7 – 315.2	305.5	+1.24
	III	316.1 – 346.1	327.4	6.6	II	400.6 – 413.8	429.0	+1.75
**LF3**	I	82.4 – 106.3	82.4	34.5	I	28.0 – 50.4	51.6	+1.43
	II	309.9 – 348.9	327.7	6.3	II	97.1 – 114.2	104.2	-27.7
**P**	I	94.8 – 143.8	110.4	32.6	I	82.7 – 101.4	99.9	+24.6
	II	221.2 – 282.6	231.2	14.7	II	291.3 – 315.5	334.1	+15.2
	III	426.6 – 436.1	412.3	9.6	III	401.5 – 455.4	453.5	+11.2
